# Third wave of African swine fever infection in Armenia: Virus demonstrates the reduction of pathogenicity

**DOI:** 10.14202/vetworld.2018.5-9

**Published:** 2018-01-11

**Authors:** M. A. Sargsyan, H. E. Voskanyan, E. M. Karalova, L. H. Hakobyan, Z. A. Karalyan

**Affiliations:** 1 Department of Epizootiology and Parasitology, Armenian National Agrarian University, Yerevan 0009, Armenia; 2 Laboratory of Cell Biology and Virology, Institute of Molecular Biology of The National Academy of Sciences of the Republic of Armenia (NAS RA), 7 Hasratyan St., Yerevan 0014, Armenia; 3 Department of Biology, Yerevan State Medical University, Yerevan, Armenia

**Keywords:** African swine fever, chronization, new isolate, viremia

## Abstract

**Aim:**

First cases of clinically uncommon African swine fever (ASF), caused by virus genotype II are described in this article. These cases occurred in Armenia, Tavush region, Dilijan municipality in 2011. The aim of this study was to identify and describe the new pathogenic forms of ASF in Armenia.

**Materials and Methods:**

The isolation and identification of ASF virus (ASFV) were carried out using conventional techniques. Clinical signs of infection were recorded daily. Gross anatomical pathology characteristics were observed during routine postmortem examinations. Blood and serum were obtained by puncture of the jugular vein using a vacutainer system.

**Results:**

The presence of ASFV DNA in the spleens was confirmed by polymerase chain reaction. Sequenced sections of p72 showed phylogenetic identity to genotype 2. The pathology exhibits unusual manifestations of the main disease. The unusual form of ASF demonstrates characteristics of a subacute form of the disease, with the possibility of conversion to a chronic form. Decreased lethality, low level of hemorrhages, and absence of severe pancytopenia in smears from spleen, lymph nodes, and blood are common features of the new form of ASF. Unlike severe thrombocytopenia in the typical ASF, the unusual form exhibited moderate or minor decrease of this feature. Despite a moderate decrease in hemadsorption titers, the unusual pattern of the disease was characterized by viremia and the presence of the virus in the visceral organs, including the brain.

**Conclusion:**

Our data allow assuming that new nosological form of ASF (genotype II) may present as a transitional form of the disease with the possibility of chronization.

## Introduction

African swine fever (ASF) is the main threat to the porcine industry in the world. Depending on viral and host factors, ASF virus (ASFV) infection of domestic swine can be expressed in several disease forms, ranging between highly lethal (up to 100%) and subclinical. Manifestations in 2007 ASF affecting domestic pigs and wild boars have been reported in the Caucasus region for the first time. The virus strain involved was related to isolates of genotype II. Almost all cases of ASF caused by genotype II can be described as peracute, acute, and/or subacute forms [1].

Initially, ASF cases in Dilijan district were recorded in 2007, while the first cases of atypical ASF in Armenia, in Dilijan district, evolved in 2011 [2]. During the first epidemic wave, hundreds of pigs were affected and eliminated from different farms. The second epidemic wave in the same region was detected in 2009 with the number of infected pigs exceeding the previous epidemic wave. The third epidemic wave was recorded in the same region in 2011. In the period from late autumn to early winter 2011 along with the typical forms of the disease were detected isolated cases of atypical course of ASF. First cases were reported in Dilijan municipality in Taush province (North-East of Armenia). All cases of atypical ASF were recorded in several farms from November 29 to December 18, 2011 [2]. Atypical ASF was observed in over 70 animals. Postmortem investigations and laboratory studies were conducted in the Institute of Molecular Biology (IMB), Armenia. ASFV sample that has been obtained from infected pigs was referred to as Dilijan 2011 IMB.

It is well known that transmission of ASF virus can occur through direct contact between sick and healthy pigs or by contact with infectious excretions and secretions. Indirect transmission can also occur if healthy animals ingest infected meat products or have contact with contaminated fomites [3]. In the context of virus transmission, chronization of disease increases risks of its transmission. First cases of clinically uncommon ASF, caused by virus genotype II, and characterized by chronization of disease, are described in this article. These cases occurred in Armenia, Tavush region, Dilijan district in 2011. Scientific investigations of the new nosological form of ASFV started in 2014 after the conclusion of an agreement with the Armenian National Agrarian University, which owned the primary material. Partial genome sequencing was performed in 2015, and several gene sequences were completed. The aim of this study was to identify and describe the new pathogenic forms of ASF in Armenia.

## Materials and Methods

### Ethical approval

Biological samples collection was approved by the Institutional Review Board/Independent Ethics Committee of the Institute of Molecular Biology of NAS RA (reference number IRB00004079).

### Sample collection

Biological samples were collected on November 30, 2011, and December 15, 2011. The investigated pigs were free from known porcine viral diseases and vaccinated against the classical swine fever. Blood and serum were obtained by puncture of the jugular vein using a vacutainer system. Samples of liver, brain, bone marrow, heart, kidney, spleen, lymph nodes, and lung were taken and held in separate, disposable plastic containers.

Clinical signs of infection were recorded daily. Gross anatomical pathology characteristics were observed during routine postmortem examinations.

### Laboratory analysis

Laboratory analyses were conducted at the Laboratory of Cell Biology and Virology, IMB of National Academy of Sciences of Armenia. All stages of processing and sample preparation for the diagnosis of ASF were performed in a biosafety level 3 laboratory by qualified staff. Blood clots from dead pigs were used to obtain serum samples, and kidney, lung, brain, bone marrow, liver, spleen, and lymph node biopsies were used for tissue samples.

DNA was extracted from 200 μl of heparinized whole blood samples and spleen samples by using a 5 PRIME Archive Pure DNA Cell/Tissue kit. Specific oligonucleotide primers and the fluorogenic probe were designed to target a highly conserved region within the B646L (p72) and B602L (chaperon) open reading frames. For phylogenetic analysis of ASFV, a portion of the highly conserved p72 gene was amplified and sequenced. The amplified products were characterized by nucleotide sequencing and compared to those obtained from published sequences.

## Results

Before third epidemic wave in Armenia, all ASF cases were documented as well-known peracute (rare) and acute and/or subacute (typical) forms [4]. Isolated cases of atypical course of ASF were detected along with typical forms of the disease in the period between late autumn and early winter 2011 in Dilijan. All cases of atypical ASF were recorded only on several from November 29 to December 18, 2011. First, the aged sows were affected than young piglets (up to 3 months old).

Table-1 presents data of laboratory tests of pigs infected by Georgia 2007 and Dilijan 2011 IMB. Studies of clinical manifestations of typical ASF (Georgia 2007) and unusual ASF (Dilijan 2011 IMB) found that unusual ASF developed obviously attenuated course of the disease. It manifested in slowed down formation of petechial hemorrhages, less intense fever, elongation of the disease duration, and development of chronic forms of pathology. The presence of ASFV DNA in the spleens was confirmed by polymerase chain reaction (PCR). We compared the nucleotide sequences obtained from the p72-based PCRs with those of previously described representative isolates. The Dilijan 2011 IMB ASFV clustered, as expected, within p72 genotype II. It showed 100% nucleotide identity with all compared ASFV circulating in the Caucasus regions since 2007.

**Table-1:** Main clinical characteristics of typical and unusual ASF.

Main indices	Typical ASF	Unusual ASF
Petechial hemorrhages of the skin	Extensive petechial hemorrhages (first arouse 23 dpi) of the skin, especially over the ears, flanks, back, ventral areas of the thorax, and abdomen. On the skin, single hemorrhage shows a tendency to fuse (indicated on 35 dpi). Hemorrhages color varies from dark pink to dark violet	Extensive petechial hemorrhages (first arouse 78) of the skin, especially over the ears, back, and ventral areas (indicated on 911 dpi). In about 60%, petechial hemorrhages were absent Hemorrhages color usually is pink and sometimes violet
Body temperature	Usually ranged between 39°C and 41.1°C, in some animal increased up to 4042°C	Moderate fever, irregular, or absent
Behavior	Anorexia and depression	Getting together, anorexia and depression on the late stage of disease
Nasal discharge	Serous, mucoid, and/or purulent	Not purulent
Digestive tract symptoms	Vomiting (in rare cases), bloody diarrhea (in about 3050%)	Absent
First symptom	Increased temperature, loss of appetite	Moderately increased temperature, lack of appetite
Other symptoms	Shallow and rapid respiration, ataxia	Ataxia
Disease duration	57 days	1112 days
Death	Usually at 7^th^ day postinfection; in about 2030% cases at 5 dpi	Usually after 710 days after arising of the first symptoms (about 2 weeks postinfection)
Lethality	100%	About 9095%. Chronization in rare cases
Myelogram	Severe lymphopenia. Decrease in the number of mature lymphocytes the number of immature immune cells, particularly myelocytes increased	Moderate lymphopenia with slight left shift. Light leukocytosis with slight left shift
Splenogram	Severe lymphopenia. Decrease in the number of mature lymphocytes, the number of immature immune cells, particularly myelocytes increased	Moderate lymphopenia, increased number of immature immune cells
Lymphogram	Severe lymphopenia. Decrease in the number of mature lymphocytes the number of immature immune cells, particularly myelocytes increased. Erythroblastosis	Absence of lymphopenia, increased number of immature immune cells. Erythroblastosis
Blood	Lymphopenia. Decrease in the number of mature lymphocytes the number of immature immune cells, particularly myelocytes increased. Severe thrombocytopenia. Erythroblastosis	Moderate leukocytosis with slight left shift. Moderate or minor thrombocytopenia. Erythroblastosis
PCR confirmed presence	All organs including head and brain	All organs including head and brain
ASFV titers in porcine sera	Positive, 4.05.5 HAD 50/mL	Positive, 2.53.0 HAD 50/mL

PCR=Polymerase chain reaction, ASFV=African swine fever virus

Postmortem examination at autopsy of the pigs infected by Georgia 2007 and Dilijan 2011. IMB isolates revealed that infection with new isolate leads to a reduction in both the number and size of hemorrhages in all visceral organs. However, swelling of the spleen and liver still persisted. Most of the lymph nodes were not affected. Specifically, unusual ASF was characterized by insignificant lymphopenia in the blood, bone marrow, and spleen. In lymph nodes, lymphopenia was absent. In all studied tissues, leukocyte population demonstrated a slight left shift. It is also necessary to state about marked (approximately 1.5-2.0 log) reduction of ASFV hemadsorption titers (compared to Georgia 2007 isolate) in porcine sera, despite the verified finding of virus in all the viscera of pigs.

**Figure-1:**
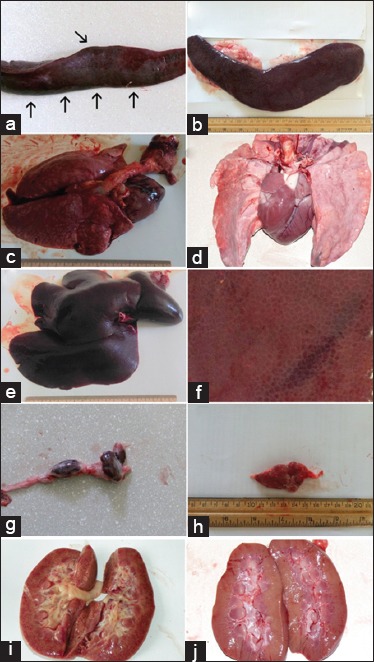
Comparative gross anatomical pathology. Left column - African swine fever virus (ASFV) Georgia 2007, right column - ASFV Dilijan 2011 Institute of Molecular Biology (IMB). (a) Spleen with hemorrhages (shown by arows) Georgia 2007. (b) Enlarged spleen without hemorrhages, infection by Dilijan 2011 IMB strain. (c) Heart and lungs with massive hemorrhages Georgia 2007. (d) Lungs with partial infarcts and point hemorrhages, infection by Dilijan 2011 IMB strain. (e) Liver with confluent hemorrhage, Georgia 2007. (f) Singe minor hemorrhages (shown by arows) Dilijan 2011 IMB. (g) Lymph nodes at 6 dpi infection by Georgia 2007 strain. (h) Lymph nodes at 10 dpi infection by Dilijan 2011 IMB strain. (i) Petechiated kidney at 6 dpi infection by Georgia 2007 strain. (j) Kidney almost without hemorrhages, at 10 dpi infection by Dilijan 2011 IMB strain.

Figure-1 shows comparative gross anatomical pathology in pigs infected by Georgia 2007 and Dilijan 2011 IMB. The spleen was enlarged and showed multiple hemorrhages in infection with Georgia 2007 (Figure-1a, arrowed) and Dilijan 2011 IMB isolate caused usually the only enlargement of the spleen (Figure-1b). Massive infarctions of the heart and lungs of animals infected by Georgia 2007 isolate were found (Figure-1c), and infection with Dilijan 2011 IMB manifested minor infractions in the lungs (Figure-1d). Hemorrhages in the liver were very common in infection with Georgia 2007 isolate and sometimes could develop generalized forms with confluent hemorrhage (Figure-1e). This pathology was not observed in infection produced by Dilijan 2011 IMB isolate, presenting with single and limited hemorrhages (Figure-1f, arrowed). There were common massive hemorrhages in the majority of lymph nodes in infection with Georgia 2007 (Figure-1g), while hemorrhages in lymph nodes developed only in few cases of Dilijan 2011 IMB infections (Figure-1h). Kidney studies showed that Georgia 2007 isolate caused multiple hemorrhages (Figure-1i), sometimes with the development of confluent hemorrhage, and Dilijan 2011 IMB isolate caused either single point hemorrhages (Figure-1j) or this pathology absent.

## Discussion

In general, the atypical form of ASF (caused by Dilijan 2011 IMB isolate) described above demonstrates the characteristics of a subacute form of the disease, with the possibility to conversion to a chronic form. The new isolate developed an uncommon clinical manifestation of ASF - unlike the common subacute form, it demonstrated partially reduced the ability for hemadsorbtion and decreased frequency and intensity of hemorrhages. It also showed a less pronounced pathology of blood clotting system compared to the typical ASF induced by Georgia 2007 ­isolate [5]. Leukopenia which is common for disease produced by Georgia 2007 isolate is less pronounced and in the majority of cases presents in lymphopenia pattern.

Presumably, such phenomenon can be either a result of penetration of a new type of ASF virus with lower virulence into the zone of previous ASF outbreaks or variation in the same virus, with the arising of new, less virulent mutant strain.

It is known that the ASFV is capable of changing quickly enough to result in a transition from acute/subacute disease to a chronic form. It is reported that the ASF virus is inclined to modification in the chronic form of the disease [6]. There can be several causes for the development of the chronic forms of ASF. Primarily, chronic ASF can be associated with infection by moderate-to-low virulence isolates [6]. However, it should be noted that, during all-time observations of ASF in Armenia, all sequenced genomes belonged to the Georgia 2007. In Armenia, only ASF (II genotype) occurs, which has penetrated the country in 2007 demonstrating absolute mortality rate among sick animals at the initial stages. In the beginning, the only form of ASF in Armenia was the acute form [7]. However, in 2010-2011, there were cases of disease transition into a chronic form [2]. This effect can be dose-depended phenomena [8,9]. The possibility of dose-dependent disease courses has been discussed by Pietschmann *et al*. [10]. However, authors found no indication of prolonged or chronic individual courses on low-dose infection. Trade and movement of pigs and pork products appear to have been one of major factors in ASFV dissemination[11].

In Armenia, like in all Caucasus region, the majority of the swine production (over 90%) can be classified as backyard and where very scarce information is available on pig trade patterns [12]. This type of swine production together with lack of information in combination with other factors such as the presence of wild boar populations and illegal trade of pigs and pig products contributed to the difficulties to ASF control [13]. Hence, we do not exclude the possibility of penetration in Armenia a new strain of genotype II, but the likelihood of such a scenario seems unlikely to us due to the closure of borders with Turkey and Azerbaijan, also because of the Muslim population of Iran, Azerbaijan, and Turkey who do not consume and therefore produce pork. This indicates that changes in the virulence of Armenian wild-type can be associated with some changes occurred in the genome of ASFV after introduction into Armenia. One of the important roles plays a change of main disease characteristics. The reasons of the persistence of ASFV in endemic areas, with small-scale but regular outbreaks in domestic pigs, is not well understood [14]. Unlike acute forms of ASF, chronic forms usually characterized by the absence of vascular lesions and by the presence of lesions in which bacteria are involved [15]. Moreover, as described by Arias and Sanchez-Vizcaıno [16], the chronic form of the disease can spread the virus for long periods of time, which likely plays a key role in the persistence of the disease.

Most likely cause of uncommon ASF is the change in the virus genome, which is consistent with a typical evolution pattern of viruses. Although DNA viruses have a significantly lower rate of mutations, compared to RNA viruses, their variability is still much higher than that in cellular organisms. Emergences of new, less virulent mutant of ASF virus has high importance for the survival of the virus, as the chances of its transmission are being increased. It is well documented that DNA viruses have a tendency to establish prolonged, chronic, and inapparent (latent) persistent infections [17,18]. The changes in the clinical manifestations of unusual ASF which are described in our article support this view. In addition, chronically infected pigs can remain persistently infected which may contribute to the prolonged ability for virus transmission. Thus, they have an important significance in disease persistence in endemic areas [19-21]. The development of appropriate ASF control strategies would require intensive epidemiological studies that could favor in the understanding of viral evolution in natural conditions.

In the current study, we show for the first time formation of the unusual or transient form of ASF during the third epidemic wave in Armenia.

## Conclusion

The new strain of ASF virus isolated from pigs in northeast Armenia caused distinct pathological changes (disease has turned into a more chronic infection during late autumn 2011), compared with acute form ASF (Georgia 2007). Nucleotide sequences obtained from the p72-based PCRs of Dilijan 2011 IMB virus clustered with p72 genotype II. So we can conclude arising of the unusual or transient form of ASF during the third epidemic wave in Armenia.

## Authors’ Contributions

ZAK and EMK designed the experiment. MAS, LHH, and HEV conducted the experiment. ZAK did technical writing and revision of the manuscript. ZAK and EMK prepared the manuscript. All authors have read and approved the final version of the manuscript.
